# Impact of direct transfer to critical care on mortality for patients who undergo emergency colorectal cancer resection: a national population-based cohort study in England

**DOI:** 10.1016/j.bjao.2026.100861

**Published:** 2026-10-15

**Authors:** Helen A. Blake, Adil Rashid, Leo Watton, Angela Kuryba, Jemma M. Boyle, Abigail Vallance, Lindy Berkman, David A. Harrison, James Doidge, S. Ramani Moonesinghe, Dave Murray, Jim Hill, Nicola S. Fearnhead, Jan van der Meulen, Linda D. Sharples, Kate Walker

**Affiliations:** 1 Research Department of Primary Care and Population Health, University College London, London, UK; 2 Clinical Effectiveness Unit, Royal College of Surgeons of England, London, UK; 3 Department of Health Services Research and Policy, London School of Hygiene and Tropical Medicine, London, UK; 4 Centre for Surgical Research, University of Bristol, Bristol, UK; 5 Patient and Public Involvement Representative, Royal College of Surgeons of England, London, UK; 6 Intensive Care National Audit and Research Centre, London, UK; 7 Anaesthesia and Peri-operative Medicine, University College London Hospitals NHS Foundation Trust, London, UK; 8 Anaesthetic Department, South Tees Hospitals NHS Foundation Trust, London, UK; 9 Division of Surgery, Manchester Royal Infirmary, Manchester, UK; 10 Department of Colorectal Surgery, Cambridge University Hospitals NHS Foundation Trust, Cambridge, UK; 11 Department of Medical Statistics, London School of Hygiene and Tropical Medicine, London, UK; 12 Research Department of Population, Policy and Practice, University College London, London, UK

**Keywords:** colon cancer, critical care, emergency surgery, high risk, outcomes

## Abstract

**Background:**

It is recommended that patients receiving emergency surgery and with ‘high-risk' of morbidity and mortality should be transferred directly from theatre to critical care. Using a national, population-based cohort study, we estimated the effect of direct transfer to critical care on 90-day mortality after emergency colorectal cancer resection, and the variation amongst English NHS hospitals.

**Methods:**

Patients who underwent emergency colorectal cancer resection between December 2016 and November 2019 according to Hospital Episodes Statistics, National Bowel Cancer Audit or National Emergency Laparotomy Audit were included. Patients were identified as having a direct transfer to critical care through linkage with the Intensive Care National Audit and Research Centre Case Mix Programme. Multivariable logistic regression and propensity score methods were used to measure the effect of direct transfer to critical care on 90-day mortality.

**Results:**

In total, 10,441 patients underwent an emergency colorectal cancer resection. Unadjusted 90-day mortality was higher in the direct transfer to critical care cohort (10.9%) than in patients without direct transfer to critical care (3.7%) (odds ratio [OR]: 3.16, 95% confidence interval [CI]: 2.65–3.77). There was no evidence of an association after risk adjustment (adjusted OR: 1.16, 95% CI: 0.94–1.43) or after propensity score analysis (weighted OR: 0.94, 95% CI: 0.69–1.27). For patients at high risk with a predicted mortality ≥5%, the proportion of patients with a direct transfer to critical care varied widely amongst hospitals (interquartile range 68%–92%; median 83%).

**Conclusions:**

Our findings suggest that apparent higher postoperative mortality in patients having direct transfer to critical care can be mostly explained by characteristics of patients, tumours, physiology and surgery. However, the extent to which direct transfer to critical care is used varies widely amongst hospitals. Further work to understand this variation has the potential to improve patient outcomes after emergency colorectal cancer resection.

**Editor’s key points**

• This national cohort included 10,441 patients undergoing emergency colorectal cancer resection in England. Direct transfer to critical care was assessed after emergency surgery during the years 2016–2019.
• Use of direct transfer to critical care varied substantially amongst hospitals.
• Higher initial mortality in patients transferred to critical care is mostly because of their more severe underlying health conditions.
• Patients transferred directly to critical care had a higher unadjusted 90-day mortality. This mortality difference was not evident after adjustment for measured patient and surgical risk factors.

In England and Wales, one in five patients diagnosed with colorectal cancer (CRC) presents in an emergency,^1^ most commonly owing to bowel obstruction,^2^^,^^3^ for which the main treatment option is primary surgical resection.^2^ Patients with CRC who undergo an emergency resection have a substantial risk of morbidity and mortality.^1^ This is multi-factorial, given patients who present in an emergency are more likely to be older and to have increased co-morbidities, a more advanced tumour stage, and more aggressive tumour biology.^4^^,^^5^ In addition, emergency presentations of CRC are associated with an acute deterioration in a patient’s clinical condition, resulting from dehydration, malnutrition, electrolyte disturbances and deranged physiology.^5^

The Royal College of Surgeons of England (RCSEng) has highlighted persistent deficits in perioperative care for patients having emergency surgery and with ‘high risk', including between-hospital variation in the availability and use of critical care facilities after surgery.^6^, ^7^, ^8^, ^9^ Internationally, higher ratios of critical care beds have been shown to improve outcomes for patients receiving emergency general surgery.^6^ Similarly, in the UK, hospitals with increased critical care capacity^7^ and critical care bed use^8^ are associated with lower 30-day mortality rates for patients receiving emergency general surgery. Notably, implementation of a care-bundle that included direct transfer to critical care after emergency abdominal surgery was associated with a reduction in postoperative mortality.^10^ As a result, the RCSEng recommends that patients receiving emergency general surgery and with ‘high risk', defined as patients with a predicted mortality ≥5%, should be transferred directly from theatre to critical care.^9^ Similarly, the World Society of Emergency Surgery consensus guidelines recommend determining the appropriate location for postoperative care on the basis of a validated preoperative risk score.^11^

Despite the RCSEng’s recommendation, the most recent National Emergency Laparotomy Audit (NELA) report found that one in five patients with a predicted risk of mortality ≥5% were not transferred directly to critical care after emergency laparotomy, with wide variation amongst hospitals.^12^ Although comparisons of hospitals were not adjusted for case-mix in patients, the size of the variation may suggest inequitable access to critical care for patients experiencing an emergency. Optimisation of perioperative care for patients having emergency CRC surgery is a priority to improve clinical outcomes. Using linked national population databases, the aim of this study is to better understand the characteristics of patients who are directly transferred to critical care after an emergency CRC resection and the influence of direct transfer to critical care on postoperative 90-day mortality, and to investigate variation amongst hospitals in the transfer of patients directly to critical care, accounting for differences in case-mix.

## Methods

### Data sources

This population-based cohort study for patients with CRC in England used several national datasets.

The National Bowel Cancer Audit (NBOCA) database is a prospective mandatory database of all patients aged 18 yrs or older diagnosed with CRC in England or Wales.^1^ Hospital Episode Statistics Admitted Patient Care (HES) is an administrative database which captures all inpatient admissions to English NHS hospitals, including *International Statistical Classification of Diseases and Related Health Problems, 10th Revision* (ICD-10) diagnosis codes and *Office of Population Censuses and Surveys Classification of Surgical Operations and Procedures*, *fourth revision* procedure codes.^13^, ^14^, ^15^ NELA is a prospective database that records the care received by patients in England and Wales who undergo an emergency laparotomy.^12^ Official death records from the Office for National Statistics (ONS) provided information on date and cause of death.^16^ The Intensive Care National Audit and Research Centre (ICNARC) Case Mix Programme is a prospective audit that collects data on outcomes for adult patients admitted to critical care units in England, Wales and Northern Ireland, including dates and times of critical care admissions and the location from which the patient was admitted to critical care.^17^

### Ethics and governance

This study was conducted as part of the NBOCA, which has established regulatory approval, data security and governance procedures. Because this research involves anonymised secondary data, UK National Research Ethics Committee approval has not been sought per their guidelines. The study was performed in accordance with the Declaration of Helsinki. This study was conducted and reported in accordance with the Strengthening the Reporting of Observational Studies in Epidemiology statement.

### Data linkage

Patient records in NBOCA, HES and NELA were linked using a spine linkage approach, whereby the HES database was designated the ‘spine dataset’, and NBOCA and NELA were linked to the spine. NBOCA and NELA were each linked to ICNARC to capture transfers to critical care. NBOCA, HES and NELA datasets were each linked to ONS mortality data. Linkage used deterministic rules regarding agreement on patient identifiers. The linkage approach has previously been described.^18^, ^19^, ^20^ When data items were available from multiple datasets, data were synthesised according to prespecified hierarchical rules.^18^

### Characteristics of patients, tumours, physiology and surgery

The NBOCA database provided data on age at diagnosis, sex, Eastern Cooperative Oncology Group performance status,^21^ ASA score,^22^ cancer stage (tumour, node and metastasis [TNM]),^23^ cancer site and surgical urgency. Surgical urgency is recorded in NBOCA according to the National Confidential Enquiry into Patient Outcome and Death pre-2004 classification in which urgent is defined as surgery ‘as soon as possible after resuscitation and usually within 24 hours’, and emergency as ‘immediate and lifesaving’ surgery.^24^

Patient comorbidity was defined in HES using the Royal College of Surgeons' Charlson Comorbidity score.^25^ This measures the presence of specific chronic medical conditions (excluding CRC), using ICD-10 diagnosis codes within the patient’s HES records in the year preceding their CRC diagnosis. HES was also used to provide mode of admission (elective or emergency).

The NELA database provides data on preoperative physiological and surgical measures including pulse rate, breathlessness, systolic blood pressure, sodium, urea, albumin and predicted peritoneal soiling.

### Definition of direct transfer to critical care (DTTCC) and 90-day mortality

The level of care provided to patients is categorised into four levels: level 0 represents standard care on the ward; level 1 represents enhanced care on the ward but without organ support; level 2 is a higher level of care including single organ support; and level 3 offers mechanical ventilation or multi-organ support.^20^ Level 2 care and level 3 care are collectively defined as critical care.^20^

Patients were defined as having a direct transfer to critical care (DTTCC) in either of the following instances^20^:

• They had a NELA record in which the destination after theatre was critical care (level 2 or level 3);

or

• They had an ICNARC record (linked through NBOCA, NELA or both) showing they were admitted to critical care from theatre or theatre recovery, and the date of admission to critical care in ICNARC within 1 day of the date of surgery in either NBOCA or NELA.

After reconciling data on date of death and date of surgery from NBOCA, HES and NELA according to prespecified hierarchical rules,^18^ we derived 90-day mortality as death from any cause within 90 days.

### Cohort definition

All patients eligible during the prespecified study period were included. Patients were eligible for analysis if they were in the spine dataset (HES) and underwent an emergency CRC resection in an English hospital between 1 December 2016 and 30 November 2019. Emergency CRC resection was defined as a CRC resection within 1 day of emergency admission in HES, urgent or emergency CRC resection in NBOCA, or CRC resection in NELA (all of which are emergency procedures according to NELA inclusion criteria). We excluded eight records with missing mortality information after reconciliation and 137 HES records that did not link to either NBOCA or NELA. Fig. 1 presents cohort derivation. Given the large dataset available, we focused on effect estimates and their confidence intervals, rather than hypothesis testing. Therefore, no formal sample size calculation was performed.

**Fig 1:**
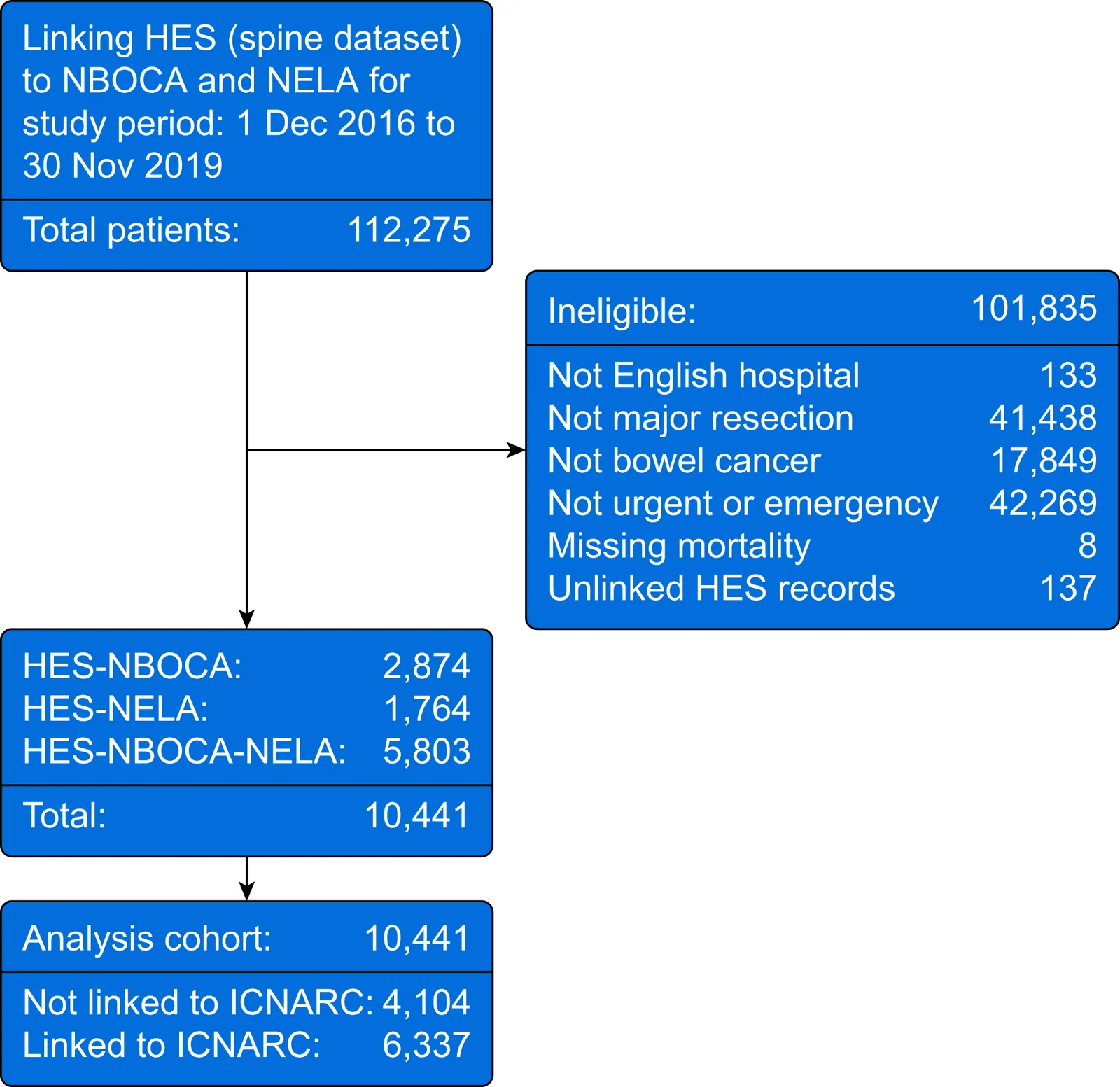
Flowchart showing inclusion criteria for the patient cohort after linkage of NBOCA and NELA to HES. HES, Hospital Episode Statistics Admitted Patient Care; NBOCA, National Bowel Cancer Audit; NELA, National Emergency Laparotomy Audit; ICNARC, Intensive Care National Audit & Research Centre Case Mix Programme.

Hospital-level care is provided by NHS ‘hospital trusts’. Hospital trusts may comprise an individual hospital or a combination of several hospitals. In this study, ‘hospital’ refers to a hospital trust.

### Statistical analysis

Characteristics and 90-day mortality of patients were compared in patients with and patients without a DTTCC.

A multivariable logistic regression model, with a random intercept for hospital, was used to estimate the effect of DTTCC on 90-day mortality. All characteristics of patients, tumours, physiology and surgery from a previously developed risk model for emergency CRC resection were included in the multivariable model.^19^ The model thus adjusted for seven preoperative physiological and surgical measures from NELA (pulse rate, systolic blood pressure, breathlessness, sodium, urea, albumin, and predicted peritoneal soiling), and 10 characteristics of patients, tumours, physiology and surgery (calendar year of surgery, age, sex, ASA physical status classification, TNM T stage, N stage, M stage, cancer site, number of comorbidities, and emergency admission). We used the same modelling strategy as the previously developed risk model^19^: sparse levels of categorical variables were combined; continuous variables were Winsorised to reduce the influence of outliers by setting observed values beyond the first and/or the 99th percentile to the value of that percentile and then modelled using fractional polynomials; and an interaction between age and metastases was included. Further details are presented in Supplementary Table 1. Missing values for the risk factors were imputed with multiple imputation using chained equations, under the missing-at-random assumption, conditional on observed data. Using the same imputation strategy as the previously developed risk model,^19^ the imputation models included the outcome and all risk factors; 20 imputed datasets were created, and Rubin's rules were used to combine the adjusted effect estimates across the datasets.^26^

Propensity score analysis, with inverse probability of treatment weighting (IPTW), was used as an alternative to multivariable logistic regression because it can more effectively balance a large number of covariates amongst treatment groups.^27^ The propensity score model included the characteristics of patients, tumours, physiology and surgery previously described. Within each imputed dataset, propensity scores were estimated and then used as weights with simple logistic regression to estimate imputation-specific weighted effects of DTTCC on 90-day mortality. Rubin's rules were then applied to these imputation-specific effect estimates to obtain a pooled effect estimate.^26^^,^^28^ Overlap of the estimated propensity scores was checked visually in each imputed dataset to assess plausibility of the positivity assumption, i.e., to confirm that there were no patients for whom DTTCC was guaranteed or impossible. Standardised differences of covariates used in the propensity score were calculated to check the balance between the exposure groups. These were used to assess the quality of the propensity score model by comparing balance after weighting (pooled across imputed datasets) with balance in the original sample with missing data.^29^

Variation in the use of DTTCC amongst hospitals was explored with and without risk adjustment for the previously mentioned characteristics of patients, tumours, physiology and surgery. First, a visual comparison was provided using funnel plot methods.^30^ Second, multilevel models with a random intercept for hospital were used to quantify the proportion of the variation in use of DTTCC because of between-hospital variation, using the intraclass correlation coefficient, with and without risk adjustment.

As a sensitivity analysis, adjusted effects were estimated separately for patients at high risk, defined as ≥5% predicted risk of mortality according to the risk model developed specifically for emergency CRC resection.^19^

Statistical analyses were conducted using Stata version 17.0 (StataCorp. 2021. *Stata Statistical Software: Release 17*. College Station, TX: StataCorp LLC.).

## Results

A total of 10,441 patients in 139 hospitals underwent an emergency CRC resection between December 2016 and November 2019 (Fig. 1). Across 10,441 patients, 58.5% (6113/10,441) had a DTTCC after emergency CRC resection. Of these, 71.8% (4387/6113) had a DTTCC according to both ICNARC and NELA (Supplementary Table 2).

Characteristics of patients and tumours according to postoperative destination are described in Table 1.^19^ Patients with a DTTCC were more likely to be at least 75 yrs old (44.6% *vs* 28.7%) and have an ASA physical status classification of 3 or higher (58.0% *vs* 30.1%) than were patients without a DTTCC. Similarly, patients with a DTTCC were more likely to have at least one comorbidity (55.6% *vs* 40.5%) and a stage T4 tumour (59.0% *vs* 48.3%) than patients without a DTTCC.

**Table 1:** Characteristics of patients and tumours according to whether patients had a DTTCC. DTTCC, direct transfer to critical care; CRC, colorectal cancer; TNM, tumour, node and metastasis. ∗At least 5% predicted risk of mortality according to a risk model developed for patients who undergo emergency CRC resection.^19^

	Had DTTCC			Did not have DTTCC		
	n	%	Mortality	n	%	Mortality
Total	6113	100.0		4328	100.0	
Died within 90 days of surgery	665	10.9		161	3.7	
Age (yrs)						
< 50	434	7.1	4.1%	527	12.2	1.5%
50–59	712	11.6	5.9%	763	17.6	2.6%
60–74	2237	36.6	9.7%	1796	41.5	3.2%
75–84	2000	32.7	13.6%	973	22.5	5.0%
≥85	730	11.9	15.9%	269	6.2	10.0%
Sex						
Female	2916	47.7	10.9%	2111	48.8	3.3%
Male	3197	52.3	10.9%	2217	51.2	4.4%
ASA physical status classification						
1	401	6.6	2.5%	608	14.5	1.2%
2	2142	35.4	4.2%	2324	55.4	2.0%
3	2597	43.0	11.4%	1124	26.8	6.3%
4 or 5	903	14.9	28.7%	139	3.3	22.3%
Missing (% of total)	70 (1.1)			133 (3.1)		
TNM T category						
T1–T2	197	4.4	7.1%	351	9.8	1.1%
T3	1647	36.6	7.2%	1493	41.9	3.1%
T4	2654	59.0	9.8%	1721	48.3	3.8%
Missing (% of total)	1615 (26.4)			763 (17.6)		
TNM N category						
N0	1827	40.7	8.4%	1487	41.8	2.8%
N1	1479	33.0	8.0%	1147	32.2	2.4%
N2	1182	26.3	9.9%	925	26.0	5.2%
Missing (% of total)	1625 (26.6)			769 (17.8)		
TNM M category						
M0	3610	83.1	8.0%	2909	83.8	2.9%
M1	732	16.9	10.7%	561	16.2	5.5%
Missing (% of total)	1771 (29.0)			858 (19.8)		
Cancer site						
Appendix/caecum/ascending colon	1610	33.1	9.8%	1473	38.7	3.5%
Hepatic flexure	271	5.6	9.2%	234	6.1	5.1%
Transverse colon	520	10.7	10.0%	396	10.4	3.3%
Splenic flexure/ descending colon	640	13.2	8.9%	407	10.7	2.2%
Sigmoid colon	1359	27.9	8.2%	855	22.4	3.3%
Rectosigmoid/rectal	466	9.6	6.9%	446	11.7	3.8%
Missing (% of total)	1247 (20.4)			517 (11.9)		
Comorbidities						
0	2612	44.3	6.4%	2440	59.5	2.0%
1	1966	33.3	11.3%	1159	28.3	3.5%
2+	1316	22.3	17.9%	499	12.2	8.2%
Missing (% of total)	219 (3.6)			230 (5.3)		
Emergency admission						
No	1234	20.2	6.1%	1582	36.6	2.7%
Yes	4876	79.8	12.1%	2742	63.4	4.3%
Missing (% of total)	3 (< 0.1)			4 (0.1)		
High risk∗	3378	55.3	17.6%	861	19.9	12.5%

For patients who had a DTTCC, 10.9% (665/6113) died within 90 days of emergency CRC resection. This compares with 3.7% (161/4328) in patients who were not directly transferred to critical care.

Table 2 shows that the unadjusted odds of 90-day mortality were approximately three times higher in patients who had a DTTCC than in patients who did not (odds ratio [OR]: 3.16, 95% confidence interval (CI): 2.65–3.77). After risk adjustment, there was no longer statistical evidence of a difference in 90-day mortality between the two groups (adjusted OR: 1.16, 95% CI: 0.94–1.43), with a wide CI that is compatible with an increase or a decrease in mortality. When using IPTW, the point estimate moved in the direction of benefit from DTTCC, but there was no statistical evidence of a difference (weighted OR: 0.94, 95% CI: 0.69–1.27; Table 2). There was good overlap in propensity scores between the DTTCC and no-DTTCC groups across all imputed datasets (Supplementary Fig. 1), and there were no propensity score values close to zero (range: 0.067–0.997). Although some propensity scores were close to 1, these extreme propensity scores were present in both exposure groups. Since IPTW will down-weight high propensity scores in the DTTCC group and up-weight those in the no-DTTCC group, any residual bias in estimates should be small. The observed covariates were well balanced after weighting, supporting the adequacy of the propensity-score model for measured confounding variables (Fig. 2, Supplementary Table 3). Residual confounding from unmeasured factors remains possible.

**Table 2:** Estimates of the association between direct DTTCC and 90-day postoperative mortality after multiple imputation, and for the subgroup with predicted 90-day mortality risk greater than 5%. CI, confidence interval; DTTCC, direct transfer to critical care; OR, odds ratio. ∗Simple logistic regression, no adjustment. ^†^Multilevel multivariable logistic regression, adjusted for 17 characteristics of patients, tumours, physiology and surgery. ^‡^Logistic regression using inverse probability of treatment weighting, where propensity score model includes 17 characteristics of patients, tumours, physiology and surgery.

Analysis	OR	95% CI
Crude OR∗	3.16	(2.65–3.77)
Adjusted OR from multivariable logistic regression^†^	1.16	(0.94–1.43)
**Sensitivity analysis: propensity score analysis**		
Weighted OR from propensity score analysis^‡^	0.94	(0.69–1.27)
**Sensitivity analysis: ≥5% predicted risk**		
Crude OR∗	1.49	(1.19–1.85)
Adjusted OR from multivariable logistic regression^†^	0.98	(0.76–1.26)

**Fig 2:**
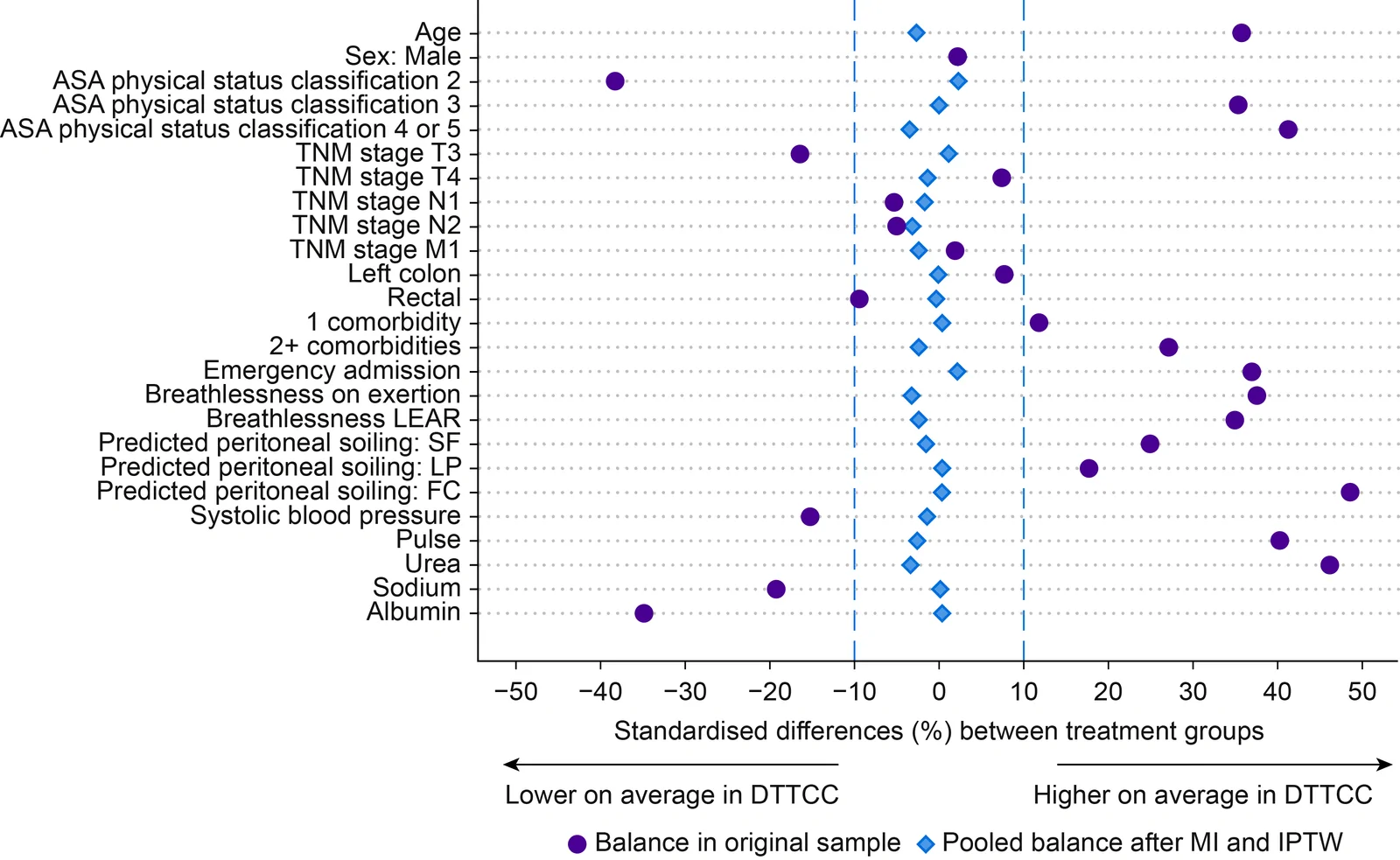
Love plot showing balance in characteristics of patients and tumours in the original sample (purple dots) and after propensity score weighting (blue diamonds). Notes: Tumour, node and metastasis (TNM) stage categories—T3, beyond muscle layer. T4, breached outer lining or invaded adjacent organs. N1, 1–3 nodes involved. N2: 4 or more nodes involved. M1, metastases. Breathlessness abbreviation—LEAR, limiting exertion and at rest. Predicted peritoneal soiling abbreviations—SF, serous fluid; LP, localised pus; FC, free pus, blood or bowel contents. Left colon: cancer site = Splenic flexure/ descending colon or Sigmoid colon. Rectal: cancer site = Rectosigmoid/Rectal Statistical abbreviations—MI, multiple imputation, IPTW: inverse probability of treatment weighting.

The proportion of patients with a DTTCC after emergency CRC resection varied substantially amongst the 139 hospitals, with an interquartile range of 44.6%–72.4%. Fig. 3 shows this wide between-hospital variation, both without and with risk adjustment for characteristics of patients, tumours, physiology and surgery. If differences amongst hospitals arose from random chance alone, the expected number of hospitals outside the 95% and 99.8% funnel limits would be 7.0 and 0.3, respectively. The observed number of hospitals outside the limits was 81 and 52, respectively, after risk adjustment.

**Fig 3:**
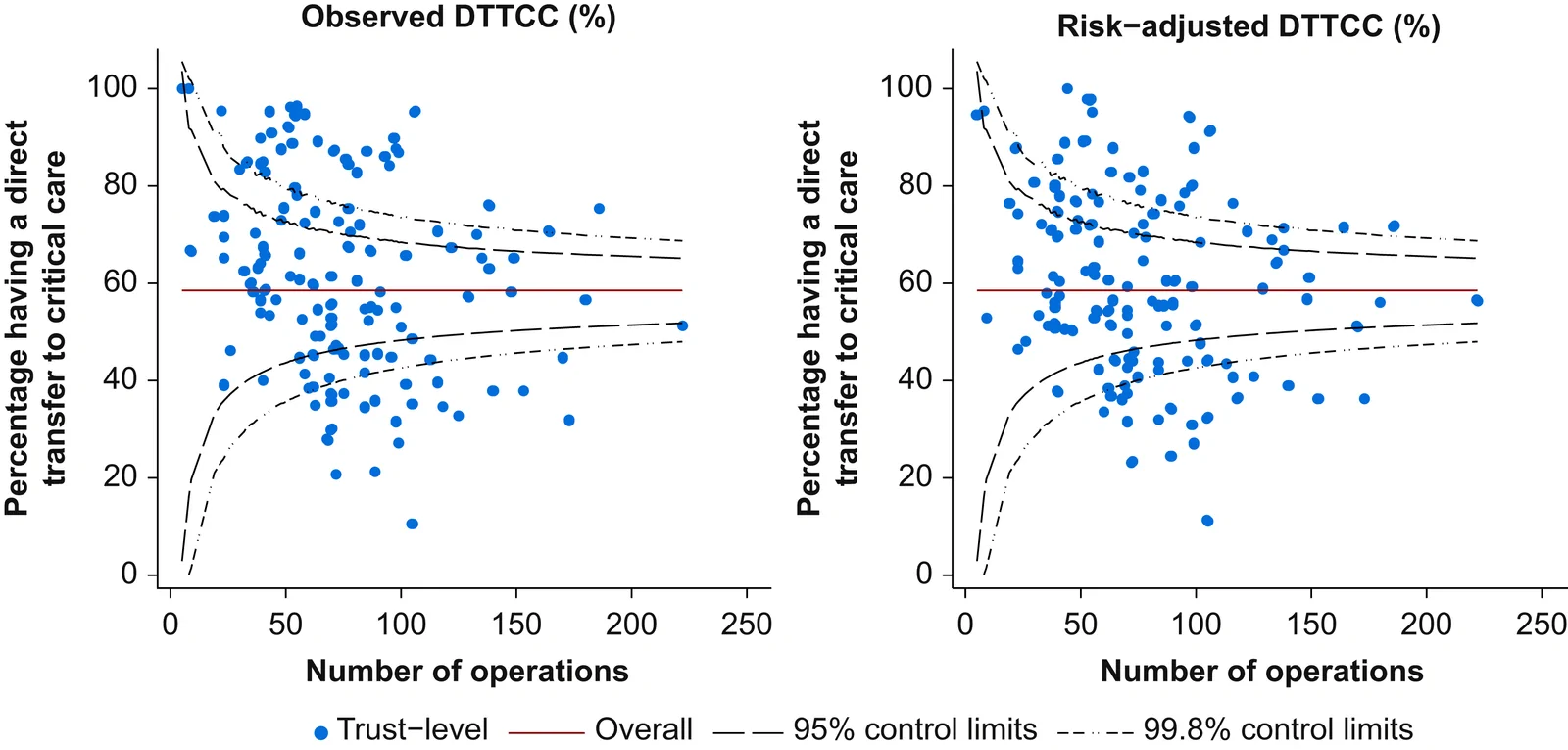
Funnel plots showing observed and risk-adjusted hospital percentages of patients directly transferred to critical care after emergency CRC resection. CRC, colorectal cancer. DTTCC, direct transfer to critical care

The intracluster correlation coefficient was 0.22 (95% CI: 0.17–0.27) without risk adjustment and 0.28 (95% CI: 0.23–0.34) with risk adjustment, indicating that, after accounting for patient, tumour, physiological and surgical factors, there remained considerable unexplained variation in the proportion of patients with a DTTCC.

Of the overall cohort, 40.6% (4239 of 10,441) were defined as being at high risk (≥5% predicted mortality risk). Of these, 79.7% (3378 of 4239) had a DTTCC. The unadjusted odds of 90-day mortality were higher in patients at high risk who had a DTTCC than in patients who did not (OR: 1.49, 95% CI: 1.19–1.85; Table 2). After risk adjustment, there was no longer statistical evidence of a difference in 90-day mortality for patients at high risk who had a DTTCC compared with patients who did not (adjusted OR: 0.98, 95% CI: 0.76–1.26). Amongst patients with ≥5% predicted risk, the variation in proportion of patients with a DTTCC after emergency CRC resection was again substantial (interquartile range: 68.2%–91.9%; Supplementary Fig. 2).

## Discussion

This national population-based study showed that six in 10 patients receiving emergency CRC resection are transferred directly to critical care after surgery, with wide variation in practice amongst hospitals, even after risk adjustment or when restricting to patients at high risk. Our findings suggest that apparent higher postoperative mortality in patients having a DTTCC can be mostly explained by characteristics of patients, tumours, physiology and surgery. The greater prevalence of established preoperative risk factors amongst patients transferred directly to critical care suggests that clinicians incorporated recognised markers of risk into postoperative destination decisions. Before risk adjustment, patients with a DTTCC had three times the postoperative mortality of patients without a DTTCC. After risk adjustment, there was no statistical evidence of an association between mortality and being transferred directly to critical care, with wide CIs compatible with an increase or a decrease in mortality.

For risk adjustment, variables from a previously developed risk model for 90-day mortality, specifically for patients who underwent emergency CRC resection, were used. These variables are predictive of postoperative mortality and include characteristics of patients, tumours, physiology and surgery.^19^ In line with previous studies, patients who had DTTCC were older and had a higher ASA physical status classification and a greater number of comorbidities, suggesting patients at high risk were appropriately selected for DTTCC. This study also highlighted that patients with a DTTCC were more likely to have locally advanced CRC.

Guidelines recommend that postoperative critical care admission should be routine for patients receiving emergency surgery and at high risk, defined as patients with a predicted hospital mortality ≥5%.^9^ According to the 90-day mortality risk model used here (based partially on the easy-to-use NELA risk model commonly used in clinical practice^31^), four in five patients with risk ≥5% have a DTTCC but with wide variation amongst hospitals (interquartile range 68–92%, median 83%). Although most patients at high risk have a DTTCC, a substantial proportion are not receiving the recommended standard of care, and the variation amongst hospitals is concerning. The reasons for this are probably multifactorial. There may be clinical uncertainty regarding whether a critical care admission would prove beneficial, based on the anticipated need for organ support or enhanced monitoring, the complexity of the major resection, and the patient's health status.^32^ In addition, unanticipated surgical findings, or dynamic intraoperative improvements in the physiology of a patient at high risk, which are not captured in this study, may lead to a decision that the patient does not require the additional support offered by critical care.

An alternative explanation may be availability of critical care capacity. Within the UK, the capacity of critical care units varies across geographical regions.^33^ Between-hospital variation in the availability of postoperative critical care facilities may contribute to deficits and inequalities in perioperative care for patients receiving emergency surgery.^9^^,^^34^ In the UK, the number of critical care beds per 100,000 people is 7.3, which is substantially lower than the Organisation for Economic Co-operation and Development European Union average of 15.9 beds.^35^

### Limitations and strengths

One limitation of this study is that despite risk adjustment with a validated model designed specifically for the prediction of postoperative mortality in patients who undergo emergency CRC resection, not all information about a patient’s physiological status is captured. As a result, full adjustment for all differences between the DTTCC and no-DTTCC cohorts may not be possible. For example, patients requiring DTTCC may be more likely to have experienced intraoperative complications, increased blood loss and increased operative duration, and to have had higher vasopressor requirements or undergone more extensive surgical resections.^36^^,^^37^ Our datasets did not include information on duration of the procedure, intraoperative instability, intraoperative complications, vasopressor requirement and blood loss; these unmeasured variables may cause an underestimate of the true effect of DTTCC. However, given these factors lie on the clinical pathway between preoperative risk and subsequent DTTCC, they can be considered mediators of the relationship between risk and outcome and may explain some of the reasons for management decisions. Further work should be performed to understand the causal pathway between preoperative risk, intraoperative mediators and DTTCC.

We assumed that if patients were admitted to critical care after their emergency CRC resection, a record should be present in ICNARC. However, although ICNARC reliably captures level 3 critical care, not all level 2 critical care units participate.^38^ Furthermore, there was incomplete linkage of the spine dataset (HES) to NELA. These limitations may each cause misclassification of DTTCC and thus bias results.^39^ However, using both NELA and ICNARC to define DTTCC, any resulting incomplete capture of DTTCC is unlikely to explain all the variation amongst hospitals.

We assumed that a 1-day difference between the date of surgery and admission to critical care reflected a direct transfer. Such discrepancies are feasible in the emergency surgery setting, where surgeries can happen at any time of day. The additional criterion of destination after surgery (destination after theatre in NELA, admission from theatre or theatre recovery in ICNARC) minimises the risk of mismeasurement bias due to inclusion of indirect transfers.

Differences in hospital critical care capacity were not captured, which may influence the timing or decision of transfer to critical care and help to understand variation in use of DTTCC amongst hospitals.^34^^,^^40^

A further limitation of using routinely collected national records concerns missing data. Record linkage can help to 'fill in' some of these gaps but can also produce missing data or selection bias owing to incomplete linkage.^39^ Indeed, in this study, there were large amounts of missing TNM staging information (available only from NBOCA) and physiological measures (available only from NELA). However, the proportion of patients with missing variables was comparable in the DTTCC and no-DTTCC cohorts, and a multiple imputation model including all risk factors and the outcome was used to maximise the analysis cohort under the missing-at-random assumption.

In general, risk scores do not predict risk perfectly, and we cannot absolutely rule out misclassification of patients at high risk. However, when risk scores are used in combination with clinical judgement, they make an important contribution in prioritising those with greatest need.

### Implications for clinical practice and future research

There is wide variation amongst hospitals in the proportion of patients being transferred directly to critical care, including amongst patients at high risk. This may reflect differences in critical care capacity or organisational structure between hospitals. It may also reflect differences in clinical policy or decision-making amongst hospitals. Further research is needed to understand and address the reasons for this variation, including investigation of hospital-level factors, such as critical care capacity, hospital volume, and organisational characteristics. In addition to quantitative research, qualitative studies should be undertaken to better understand differences in critical care use.

Our work also has important implications for countries with similar health services. Further work should be conducted in other countries to further understand factors determining critical care capacity and decision-making.

## Conclusion

For patients with colorectal cancer (CRC) having emergency resection, differences in postoperative mortality for those with a direct transfer to critical care can be mostly explained by characteristics of patients, tumours, physiology and surgery. Across England as a whole, most patients at high risk with CRC are being transferred directly from theatre to critical care. A key finding for clinical practice is that there is wide variation amongst hospitals in the direct transfer of patients with CRC to critical care after emergency resection, even after careful risk adjustment, and when restricting to patients at high risk. Further research is urgently required to understand the underlying causes of this variation, enabling targeted strategies to improve practice and ensure consistent delivery of guideline-based postoperative care.

## Authors’ contributions

Data curation, methods, data analysis and interpretation, data visualisation, writing – original draft, review and editing: HAB

Data interpretation, writing – original draft, review and editing: AR

Data interpretation, writing – original draft, review and editing: LW

Data interpretation, writing – review and editing: AK

Data interpretation, writing – review and editing: JMB

Data interpretation, writing – review and editing: AV

Patient and public involvement and engagement, data interpretation, writing – review and editing: LB

Data interpretation, writing – review and editing: DAH

Data interpretation, writing – review and editing: JD

Data interpretation, writing – review and editing: SRM

Data interpretation, writing – review and editing: DM

Data interpretation, writing – review and editing: JH

Data interpretation, writing – review and editing: NSF

Conceptualisation, methods, data interpretation, funding acquisition, writing – original draft, review and editing: JvdM

Funding acquisition, methods, writing – original draft, review and editing: LDS

Conceptualisation, methods, data interpretation, funding acquisition, writing – original draft, review and editing: KW

## Disclaimer

The views and opinions expressed herein are those of the authors and do not necessarily reflect those of ICNARC.

## Data availability statement

The data used in this study are available on request from Healthcare Quality Improvement Partnership through their data access request process. The authors do not have permission to share the patient-level records used in the analysis.

## Funding

The National Bowel Cancer Audit is commissioned by the Healthcare Quality Improvement Partnership as part of the National Clinical Audit and Patient Outcomes Programme and funded by NHS England and the Welsh Government (www.hqip.org.uk/national-programmes). These sponsors were not involved in the study design; in the collection, analysis and interpretation of data; in the writing of the report; and in the decision to submit the article for publication. This study was funded by the National Institute for Health Research (NIHR) HS&DR Project:17/05/45. This report is independent research supported by the 10.13039/501100000272NIHR ARC North Thames. The views expressed are those of the author(s) and not necessarily those of the NIHR or the Department of Health and Social Care.

## Declarations of interest

The authors declare that they have no conflicts of interest.
