# Supplementary material for: Impact of direct transfer to critical care on mortality for patients who undergo emergency colorectal cancer resection: a national population-based cohort study in England
Source: BJA Open. 2026 Oct 15;20:100861. doi: 10.1016/j.bjao.2026.100861 (PMC13694773; doi:10.1016/j.bjao.2026.100861)
Supplement: Supplementary file 1 [file mmc1.docx]

Supplementary Material

The impact of direct transfer to critical care on mortality for patients undergoing emergency colorectal cancer resection: a national population‐based cohort study in England

Helen A Blake^1,2,3^, Adil Rashid^2,3^, Leo Watton^2,3^, Angela Kuryba^2,3^, Jemma Boyle^2^, Abigail Vallance^4^, Lindy Berkman^5^, David A Harrison^6^, James Doidge^6^, S Ramani Moonesinghe^7^, Dave Murray^8^, Jim Hill^9^, Nicola Fearnhead^10^, Jan van der Meulen^2,3^, Linda Sharples^3^, Kate Walker^2,3^

1. University College London, London, UK.
2. Royal College of Surgeons of England, London, UK.
3. London School of Hygiene and Tropical Medicine, London, UK.
4. University of Bristol, Bristol, UK.
5. Patient and Public Involvement Representative, London, UK.
6. Intensive Care National Audit & Research Centre, London, UK.
7. University College London Hospitals NHS Foundation Trust, London, UK.
8. South Tees Hospitals NHS Foundation Trust, London, UK.
9. Manchester Royal Infirmary, Manchester, UK.
10. Cambridge University Hospitals NHS Foundation Trust, Cambridge, UK

Corresponding author: Helen Abigail Blake, helen.blake@ucl.ac.uk

Supplementary Table 1: Variables adjusted for in the multivariable logistic regression model used to estimate the effect of DTTCC on 90-day mortality. The model included a random intercept for hospital. The model also included the interaction between age and TNM M stage.

| **Categorical** | **Number of** | **Reference** | | **Functional Form** |
| --- | --- | --- | --- | --- |
| **variables** | **categories** | **category** | | **(dummy variables)** |
| Year of surgery | 4 | 2016 | | 2017; 2018;  2019. |
| Sex | 2 | Female | | Male. |
| ASA grade | 4 | 1 | | 2;  3;  4 or 5. |
| TNM T stage | 3 | T1 or T2 (inner layer of bowel or into muscle layer) | | T3 (beyond muscle layer);  T4 (breached outer lining or invaded adjacent organs). |
| TNM N stage | 3 | N0 (no nodes involved) | | N1 (1-3 nodes involved);  N2 (4 or more nodes involved). |
| TNM M stage | 2 | M0 (no metastases) | | M1 (metastases). |
| Cancer site | 6 | Appendix/ caecum/ ascending colon | | Hepatic flexure;  transverse colon;  splenic flexure/descending colon;  sigmoid colon;  rectosigmoid/rectal |
| Number of comorbidities | 3 | 0 | | 1;  2+. |
| Emergency admission | 2 | No | | Yes. |
| Breathlessness | 3 | No breathlessness history | | On exertion;  limiting exertion and at rest. |
| Predicted peritoneal soiling | 4 | No predicted peritoneal soiling | | Serous fluid;  localised pus;  free pus, blood or bowel contents. |
| **Continuous** | **Range before** | **Range after** | **Functional Form** | |
| **variables** | **Winsorising** | **Winsorising** | **(fractional polynomials)** | |
| Age | 18-102 | n/a | (age - 70) + (age - 70)^2^ | |
| Pulse rate | 11-200 | 55-140 | (pulse - 85) + (pulse - 85)^2^ | |
| Systolic blood pressure | 10-238 | 80-188 | (systolic blood pressure - 130) + (systolic blood pressure - 130)^2^ | |
| Sodium | 100-176 | 124-145 | (sodium - 99)^3^ + (sodium - 99)^3^×ln(sodium - 99) | |
| Urea | 1-290 | 1-29.7 | (ln(urea) - ln(6)) + (ln(urea) - ln(6))^2^ | |
| Albumin | 0.6-96 | 16-50 | (albumin - 36) | |

Supplementary Figure 1: Propensity score overlap in each imputed dataset, comparing those who did (red) and did not (blue) have a direct transfer to critical care (DTTCC).

Supplementary Table 2: Numbers of patients defined as having a direct transfer to critical care (DTTCC) according to ICNARC, NELA, or both.

|  | DTTCC according to NELA | | |
| --- | --- | --- | --- |
| DTTCC according to ICNARC | No | Yes | Total |
| No | 4,328 | 687 | 5,015 |
| Yes | 1,039 | 4,387 | 5,426 |
| Total | 5,367 | 5,074 | 10,441 |

Supplementary Table 3: Pooled standardised mean differences for the patient and tumour characteristics, comparing the original sample versus after propensity score weighting.

|  | **Standardised differences (%)** | |
| --- | --- | --- |
| **Covariate** | **in original sample** | **pooled after MI and IPTW** |
| Age | 35.74 | -2.68 |
| Sex: Male | 2.15 | 2.22 |
| ASA grade 2 | -38.23 | 2.27 |
| ASA grade 3 | 35.34 | -0.02 |
| ASA grade 4 or 5 | 41.26 | -3.49 |
| TNM stage T3 (beyond muscle layer) | -16.43 | 1.13 |
| TNM stage T4 (breached outer lining or invaded adjacent organs) | 7.41 | -1.35 |
| TNM stage N1 (1-3 nodes involved) | -5.31 | -1.71 |
| TNM stage N2 (4 or more nodes involved) | -5.06 | -3.17 |
| TNM stage M1 (metastases) | 1.86 | -2.41 |
| Left colon | 7.67 | -0.10 |
| Rectal | -9.40 | -0.34 |
| 1 comorbidity | 11.83 | 0.36 |
| 2+ comorbidities | 27.16 | -2.41 |
| Emergency admission | 36.97 | 2.15 |
| Breathlessness on exertion | 37.55 | -3.25 |
| Breathlessness limiting exertion and at rest | 34.95 | -2.41 |
| Predicted peritoneal soiling: serous fluid | 24.90 | -1.55 |
| Predicted peritoneal soiling: localised pus | 17.71 | 0.36 |
| Predicted peritoneal soiling: free pus/blood/bowel contents | 48.52 | 0.33 |
| Systolic blood pressure | -15.21 | -1.43 |
| Pulse | 40.27 | -2.59 |
| Urea | 46.12 | -3.38 |
| Sodium | -19.23 | 0.14 |
| Albumin | -34.84 | 0.36 |

Note – This table supports Figure 2.

Abbreviations – MI: multiple imputation, IPTW: inverse probability of treatment weighting.

Supplementary Figure 2: Funnel plot (using imputed data) showing observed between-hospital variation in the percentage of patients directly transferred to critical care following emergency colorectal cancer resection. Restricted to high-risk patients (≥5%) according to the 90-day mortality risk- adjustment model.
